# A phase II trial of the vitamin D analogue Seocalcitol (EB1089) in patients with inoperable pancreatic cancer

**DOI:** 10.1038/sj.bjc.6600162

**Published:** 2002-03-04

**Authors:** T R J Evans, K W Colston, F J Lofts, D Cunningham, D A Anthoney, H Gogas, J S de Bono, K J Hamberg, T Skov, J L Mansi

**Affiliations:** CRC Department of Medical Oncology, Beatson Oncology Centre, Western Infirmary, Dumbarton Road, Glasgow G11 6NT, UK; Department of Clinical Biochemistry, St George's Hospital Medical School, Cranmer Terrace, London SW17 0RE, UK; Department of Medical Oncology, St George's Hospital, Blackshaw Road, London SW17 0QT, UK; Section of Medicine, The Royal Marsden Hospital, Downs Road, Sutton, Surrey, SM2 5PT, UK; Biological Research Department, Leo Pharmaceutical Products, DK-2750 Ballerup, Denmark; Medical Department, Leo Pharmaceutical Products, DF-2750 Ballerup, Denmark

**Keywords:** pancreatic cancer, vitamin D, hypercalcaemia

## Abstract

Inoperable cancer of the exocrine pancreas responds poorly to most conventional anti-cancer agents, and new agents are required to palliate this disease. Seocalcitol (EB1089), a vitamin D analogue, can inhibit growth, induce differentiation and induce apoptosis of cancer cell lines *in vitro* and can also inhibit growth of pancreatic cancer xenografts *in vivo*. Thirty-six patients with advanced pancreatic cancer received once daily oral treatment with seocalcitol with dose escalation every 2 weeks until hypercalcaemia occurred, following which patients continued with maintenance therapy. The most frequent toxicity was the anticipated dose-dependent hypercalcaemia, with most patients tolerating a dose of 10–15 μg per day in chronic administration. Fourteen patients completed at least 8 weeks of treatment and were evaluable for efficacy, whereas 22 patients were withdrawn prior to completing 8 weeks' treatment and in 20 of these patients withdrawal was due to clinical deterioration as a result of disease progression. No objective responses were observed, with five of 14 patients having stable disease in whom the duration of stable disease was 82–532 days (median=168 days). The time to treatment failure (*n*=36) ranged from 22 to 847 days, and with a median survival of approximately 100 days. Seocalcitol is well tolerated in pancreatic cancer but has no objective anti-tumour activity in advanced disease. Further studies are necessary to determine if this agent has any cytostatic activity in this malignancy in minimal disease states.

*British Journal of Cancer* (2002) **86**, 680–685. DOI: 10.1038/sj/bjc/6600162 www.bjcancer.com

© 2002 Cancer Research UK

## 

Carcinoma of the exocrine pancreas is the fifth commonest cancer and the fourth commonest cause of cancer deaths in the UK (Williamson, 1988). Conventional methods of treatment including surgery, radiotherapy and chemotherapy offer little hope of cure, and the 5-year survival is reported as less than 1% with a median survival of less than 3 months (Cancer of the Pancreas Task Force Group, 1981).

Cancer of the pancreas responds poorly to most single-agent or combination chemotherapy regimens. Nevertheless, palliative chemotherapy can improve overall survival compared with no treatment without impairing quality of life (Mallinson *et al*, 1980; Leonard *et al*, 1992; Palmer *et al*, 1994). Objective response rates remain low with most single-agent chemotherapy including 21–26% with 5FU (Carter, 1975; Moertel, 1976), 26% with ifosfamide (Bernard *et al*, 1986), 22% with epirubicin (Wils *et al*, 1985) and 21% with cisplatin (Wils *et al*, 1993). Furthermore, the results of combination chemotherapy have also been disappointing with objective response rates of only 26% using 5FU with BCNU (Kovach *et al*, 1974), 10% with 5FU and mitomycin C (Buroker *et al*, 1979), 14% with FAM (5FU, doxorubicin, mitomycin C) (Oster *et al*, 1986), and 17% with continuous infusional 5FU administered with epirubucin and cisplatin (Evans *et al*, 1996). More recently, gemcitabine was observed to have promising activity in phase II trials (Casper *et al*, 1994; Rothenberg *et al*, 1996). Gemcitabine has been shown to be more effective than 5FU in a randomised, controlled phase III trial, although the 5FU was administered in a sub-optimal way (Burris *et al*, 1997). However the reported objective response rate for gemcitabine was only 5.4%, with only a modest survival advantage. Consequently, the development of novel therapies in pancreatic cancer is justified.

The active vitamin D metabolite, 1,25-dihydroxyvitamin D_3_, plays an important role in calcium homeostasis. In addition, it has a role in the control of cellular differentiation and proliferation (Bell, 1985; Reichel *et al*, 1989) and can promote cellular differentiation and inhibit proliferation of cancer *in vitro* (Colston *et al*, 1981; Frampton *et al*, 1983; Brehier and Thomasset, 1988) as well as inhibiting the invasive potential of human breast cancer cells *in vitro* (Hansen *et al*, 1994) and inhibiting tumour-induced angiogenesis (Majewski *et al*, 1993).

Furthermore, 1,25(OH)_2_D_3_ can induce apoptosis in human breast cancer and leukaemic cell lines (James *et al*, 1995; Elstner *et al*, 1996). The hormone mediates its action through its nuclear receptor (vitamin D receptor, VDR) which is a transactivating transcriptional factor and a member of the steroid nuclear hormone receptor superfamily of genes (Evans, 1988). In addition, there is evidence to support the notion of another, non-genomic pathway by which vitamin D can initiate various biological responses (Norman *et al*, 1996; Nemere and Farach-Carson, 1998).

The calcaemic effect of 1,25(OH)_2_D_3_ *in vivo* limits its potential as a therapeutic agent. The synthetic analogue seocalcitol is 50–200 times more potent than vitamin D in inhibiting growth and inducing differentiation of cancer cell lines (Hansen *et al*, 1997; Kissmeyer *et al*, 1997). *In vivo* studies in animal models have shown that seocalcitol can cause regression of established tumours, prevent the development of metastases, and prolong survival time in tumour-bearing animals (Colston *et al*, 1992, 1997; James *et al*, 1998; Lokeshwar *et al*, 1999; Nickerson and Huynh, 1999), with significant inhibition of tumour progression achieved at doses that do not cause significant hypercalcaemia (Colston *et al*, 1992). Furthermore, seocalcitol inhibits growth of pancreatic cancer cells *in vitro* (Zugmaier *et al*, 1996; Pettersson *et al*, 2000) and inhibits growth *in vivo* of pancreatic cancer xenografts in immunodeficient mice (Colston *et al*, 1997). Seocalcitol was well tolerated in a phase I clinical study in patients with breast or colorectal cancer, with dose-dependent hypercalcaemica (Gulliford *et al*, 1998). Consequently a phase II study was performed in patients with inoperable pancreatic cancer to determine the objective anti-tumour activity of seocalcitol in this disease.

## MATERIALS AND METHODS

The study was a multi-centre, open, non-controlled trial at the Beatson Oncology Centre, Glasgow; St George's Hospital, London; and the Royal Marsden Hospital, London and Surrey. The study was approved by the Local Research Ethics Committee of all participating institutions, and all patients gave written, informed consent.

Eligible patients were those with histologically or cytologically confirmed inoperable carcinoma of the exocrine pancreas, age ⩾18 years, WHO performance status of 0–2, a life expectancy of at least 3 months, an albumin-corrected serum calcium of <2.65 mmol l^−1^, and adequate renal (gfr ⩾40 ml min^−1^; serum creatinine ⩾2 times the upper limit of normal), hepatic (bilirubin ⩾1.5 times the upper limit of normal) and haematological (Hb ⩾10 g dl^−1^, WBC ⩾3.0×10^9^ ^l−1^, platelets ⩾100×10^9^ ^l−1^) function. Patients with a history of hypercalcaemia, disordered calcium metabolism, anti-cancer therapy within the previous 4 weeks, or calcium-lowering therapy (including corticosteroids above the equivalent of 25 mg prednisolone daily) within the previous 2 weeks, were excluded.

The study comprised two parts: (i) a dose-finding phase to determine the individual maximum tolerated dose (MTD) for each patient; and (ii) a treatment maintenance phase to determine the response to seocalcitol and the tolerability of treatment.

### Patient treatment schedules

Patients started seocalcitol at a dose of 20 μg given as a once-daily dose just prior to the evening meal. The seocalcitol dose was increased every 2 weeks until a dose was reached that resulted in hypercalcaemia, this being defined at the beginning of the study as either a fasting albumin-corrected serum calcium of >2.80 mmol l^−1^ or non-fasting albumin-corrected serum calcium of >3.0 mmol l^−1^. During most of the study, there was no special limit for fasting serum calcium, and the dose was kept constant for serum calcium values between 2.80 and 3.00 mmol l^−1^. When a patient developed hypercalcaemia above 3.00 mmol l^−1^, seocalcitol was stopped for 1 week and then recommenced at the dose level immediately below the dose causing hypercalcaemia. Thereafter no further dose adjustment was performed unless further episodes of hypercalcaemia occurred. Dose levels of 5, 10, 15, 20, 30, 40 and 60 μg daily were allowed.

### Calcium diet

Patients were seen by a dietician prior to starting seocalcitol therapy and after 4 weeks of treatment, and dietary calcium intake assessed. All patients were given written advice on dietary calcium intake and encouraged to adhere to a ‘lowest acceptable calcium diet’ (400 mg day^−1^ of calcium) excluding all dairy products. Patients with inoperable pancreatic cancer are prone to cachexia and poor nutrition. However, it was considered justified to advise these patients to take a low-calcium diet given the *in vivo* data supporting a higher anti-tumour activity with increased seocalcitol dose (Colston *et al*, 1992). The involvement of dieticians in these assessments was an attempt to achieve a low-calcium diet without further compromising patients' nutritional status. Dietary compliance was checked at follow-up visits by individual investigators.

### Assessment of toxicity and response

Prior to starting therapy, all patients underwent clinical assessment and measurement of full blood count, serum urea, electrolytes, liver function tests, glucose, parathyroid hormone and CEA. Serum albumin, creatinine, total calcium and ionised calcium were also measured and the serum calcium corrected for albumin using the following formula: albumin corrected serum calcium=total serum calcium+[(40-serum albumin)×0.02]. Glomerular filtration rate (ml min^−1^) was calculated on an 8-h urine collection. Chest X-ray and CT scan of the abdomen (and other sites of measurable disease as appropriate) were performed up to 2 weeks prior to starting therapy. The presence and severity of the following signs and symptoms of pancreatic cancer were recorded: reflux, pain, anorexia, nausea, vomiting, change in bowel habit and dyspnoea.

The serum albumin, calcium (total), creatinine and ionised calcium were measured weekly until the serum albumin-corrected calcium had been stable for 4 weeks at the maximum intended seocalcitol dose. Thereafter samples were measured at 4-weekly intervals. The full blood count and other biochemical analyses (urea, electrolytes, glucose, parathyroid hormone) were measured at 4-week intervals throughout the study.

Toxicity was graded based upon the SWOG CTC profile (Green and Weiss, 1992) and was recorded at 4-weekly intervals throughout the study. Similarly, physical examination and assessment of the signs and symptoms of pancreatic cancer were measured at every 4 weeks throughout the study. Disease assessments by chest X-ray, CT scan of the abdomen (and other sites as at pre-treatment) were repeated after 12, 24 and 52 weeks during the study and at other times as clinically indicated. Response assessments were determined using the SWOG criteria (Green and Weiss, 1992). Patients continued seocalcitol for up to 52 weeks but were withdrawn earlier if there was evidence of disease progression or unacceptable toxicity. Treatment could be extended on a compassionate use basis for patients having response or stable disease after 1 year.

### Statistics

The number of patients for inclusion in the study was determined according to Gehan's 2-stage design for estimating the response rate (Gehan, 1961). Thus the sample size calculation was based on the requirement of stopping the study at an early stage if the response rate is below 20%, and of estimating the response rate with a standard error less than 0.10. Therefore if there was no objective tumour response (complete or partial) among the first 14 evaluable patients (patients completing at least 8 weeks of seocalcitol), recruitment of patients would stop. Patients who did not complete at least 8 weeks of seocalcitol were replaced. Additional patients would be included if there was one or more responses in the first 14 patients according to Gehan's design. Duration of stable disease was measured from the start of seocalcitol until the last date where stable disease was observed. The time to treatment failure was defined as the time from start of treatment until progression was first detected or the patient went off study due to clinical deterioration (without tumour measurements) or the need for other anticancer treatments. Overall survival was measured from the start of seocalcitol treatment until death from any cause.

## RESULTS

### Patient characteristics

A total of 43 patients were enrolled from December 1995 with the final patient completing treatment in June 1999. The mean age was 60.7 years (range 38–77), 29 were male and 14 female, 38 of Caucasian origin with three Negro and two of Oriental/Asian origin. Forty-one patients had a histological or cytological diagnosis of carcinoma. One further patient had a diagnosis of pancreatic cancer made on radiological evidence but without histological or cytological confirmation. A further patient was enrolled into the study in the belief that he had histological or cytological confirmation of pancreatic cancer but no biopsy had been performed. CT-guided biopsy of a pancreatic mass was subsequently performed and did not confirm that the lesion was malignant and the patient was then withdrawn from the study (subsequently post-mortem confirmed the diagnosis of carcinoma of the exocrine pancreas). Of the 41 patients with a confirmed diagnosis of pancreatic cancer, there was one case of a neuroendocrine cancer, one of carcinoma of unknown type, and 39 of adenocarcinoma, with differentiation classified as well (two patients), moderate (five patients), poor (four patients) and unknown (28 patients). Previous non-surgical anti-cancer treatment included palliative chemotherapy (12 patients), radiation therapy (two patients) with no patients having received prior hormonal therapy. Distant metastases were observed in 26 patients including liver (19 patients), lung (seven patients), lymph nodes (three patients) and peritoneal (one patient) metastases.

### Patient population

All patients attended for screening and investigations. However, of the intention-to-treat population, seven patients were withdrawn prior to starting therapy due to clinical deterioration (four patients), deteriorating liver function tests (one patient) and refusal to comply with diet (two patients). Thus 36 patients started seocalcitol therapy and formed the patient population that was evaluable for toxicity. Fourteen patients completed at least 8 weeks treatment and were evaluable for analyses of efficacy. Twenty-two patients were withdrawn before completing sufficient therapy for evaluation of efficacy, due to clinical disease-related deterioration (14 patients), death due to disease progression (six patients), inability to adhere to diet (one patient), and lack of confirmation of diagnosis on histology/cytology (one patient). Of the 14 patients who were evaluable for analyses of efficacy, four patients had locally advanced disease and 10 had distant metastases (liver (eight patients), liver and lung (one patient), lung and lymph nodes (one patient)).

### Calcium levels and seocalcitol dose

The individual maximum tolerated dose was defined as the highest non-calcaemic dose used, i.e. the final dose given at the end of the titration phase unless the patient subsequently experienced hypercalcaemia at that dose. The individual MTD could be determined in 29 of the 36 patients in the treated population. The remaining seven patients experienced hypercalcaemia at the final dose given and had not received lower doses previously without developing hypercalcaemia. One patient developed hypercalcaemia at the final dose given, and had previously received the dose step below without developing hypercalcaemia, and this was therefore chosen as the individual MTD.

**Figure 1:**
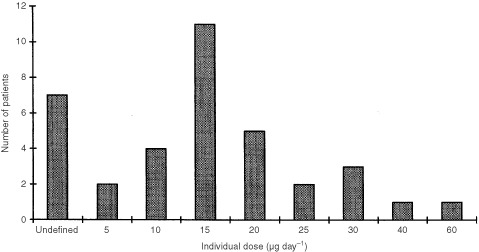
Individual MTD for patients entering the maintenance phase of the study (*n*=36).

The individual MTD for the patient population who received seocalcitol ranged from 5 to 60 μg day^−1^. However the individual MTD could not be reliably determined in seven patients who were withdrawn from the study during the titration phase for reasons not related to treatment. The individual MTD in the 29 patients who continued in the maintenance phase ranged from 5–60 μg day^−1^ and is shown in Figure 1

**Figure 2:**
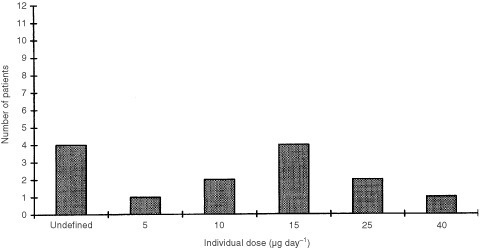
Individual MTD for patients who received at least 8 weeks of seocalcitol and who were evaluable for efficacy analyses (*n*=14).

. The individual MTD for the 14 patients who completed at least 8 weeks of treatment and who were evaluable for response, ranged from 5–40 μg day^−1^ (Figure 2

). The most common maximum non-calcaemic daily dose was 15 μg day^−1^.

### Toxicity

The most frequent toxicity was dose-related hypercalcaemia. There was a significant increase in the ratio of the end of treatment albumin-adjusted serum calcium to the corresponding baseline value of 1.13 (range 0.88–1.36; *P*<0.001, *n*=36), and similarly for the ratio of serum ionised calcium (1.11; range 0.99–1:38; *P*=0.001, *n*=36) and in the ratio for serum creatinine (1.25, range 0.67–3.59; *P*<0.001, *n*=36).

Other frequently recorded symptoms included nausea, vomiting, abdominal pain and change of bowel habit, although these symptoms could also be attributed to the underlying malignancy. Grade 3/4 toxicity as determined by the SWOG criteria occurred in only two patients with grade 4 haematological toxicity (one patient), and grade 4 metabolic toxicity (one patient). One patient developed grade 4 lowering of the lymphocyte count after 4 weeks' treatment but with a normal total WBC and normal total neutrophil count. This patient came off study shortly after this due to further biliary obstruction. The second patient developed grade 4 hypercalcaemia after 8 weeks treatment, and this resolved on stopping seocalcitol for 2 weeks, after which seocalcitol was re-started at a lower dose level.

### Efficacy analyses

There were no partial or complete responses. Of the 14 patients who received at least 8 weeks of therapy, stable disease was achieved in five patients at the first disease assessment, with documented disease progression in six patients, and clinical deterioration in keeping with disease progression in the remaining three patients. Three of the patients with stable disease at the first assessment had locally advanced disease, and two had distant metastases. The duration of stable disease in these five patients ranged from 82–532 days (median 168 days).

**Figure 3:**
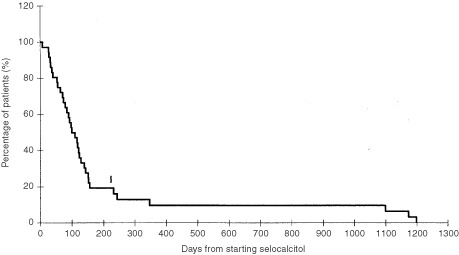
Kaplan–Meier plot of overall survival in days (all patients). The one patient remaining alive at the time of data censoring is marked with a vertical bar.

The time to treatment failure for all patients who received seocalcitol (*n*=36) ranged from 22 to 847 days (mean 120 days). The median survival (*n*=36) was approximately 100 days, with only 10% of patients surviving for more than 1 year (Figure 3

).

There was no correlation between individual dose and either time to treatment failure or overall survival.

## DISCUSSION

The most frequent toxicity of seocalcitol in advanced pancreatic cancer is dose-dependent hypercalcaemia. The acceptable daily dose in protracted administration in this study was 10–15 μg daily which is similar to the dose of 7 μg m^−2^ per day estimated to be tolerable for most patients in the phase 1 study in breast and colorectal cancer (Gulliford *et al*, 1998). The other commonly reported events were nausea, vomiting, abdominal pain and change of bowel habit. However in many cases these events could also be attributed to the underlying malignancy.

Of the 36 patients who started treatment with seocalcitol, only 14 completed 8 weeks of treatment for evaluation of efficacy analyses, with 20 patients deteriorating clinically and being withdrawn from the study prior to completing 8 weeks of treatment. There were no objective responses to seocalcitol in these 14 patients. Thus seocalcitol is ineffective as a cytotoxic agent in advanced pancreatic cancer in terms of anti-tumour efficacy as determined by a reduction in tumour volume.

Cancer of the exocrine pancreas is characterised by a natural history of rapid disease progression in patients with inoperable disease with obstructive jaundice and duodenal obstruction frequent complications. Seocalcitol can inhibit cellular proliferation, induce differentiation and induce apoptosis in cancer cell lines *in vitro*. However it is likely that it has a cytostatic rather than cytotoxic effect *in vivo*, and its optimal clinical effect is therefore most likely to be achieved in minimal disease states such as in adjuvant therapy or as maintenance therapy after chemotherapy has induced disease stabilisation. Of the 14 patients who received at least 8 weeks of seocalcitol treatment, stable disease was achieved in five patients with duration of stable disease ranging from 82 to 532 days (median duration of disease stabilisation of 168 days) which is similar to the duration of benefit in patients who respond or have disease stabilisation with chemotherapy in this disease. However, in a non-randomised phase II study, it can not be determined if this is due to the anti-tumour activity of seocalcitol or the natural history of the disease in this selected group of patients. Furthermore, in a non-randomised phase II study, time to disease progression is not a valid study endpoint in the absence of a comparator group of patients.

In conclusion, seocalcitol is safe when administered on a daily basis over several weeks in patients with advanced pancreatic cancer. No objective responses were seen. However, given the tolerable toxicity profile of this agent, and the pre-clinical evidence to suggest that it may have activity in this malignancy, further studies are required to determine if it produces a demonstrable biological effect (e.g. by functional imaging such as with PET scanning) in the absence of objective responses which would justify proceeding to subsequent randomised studies to explore the effect of seocalcitol on overall survival when given in combination with chemotherapy, or as ‘maintenance therapy’ after chemotherapy in advanced disease, or in the adjuvant setting.
